# Adulterations

**Published:** 1860-10

**Authors:** 


*Adulterations.*—We are but slowly correcting the destroying arts of tradesmen in their various adulterations. Some of the states, as Ohio and New York, have made laws and appointed Chemists for the examination of liquors, leading to many fearful discoveries in the nature of slow poisoning of all whiskey and brandy drinkers, but it must be extended to include all articles of consumption. For instance, last year, Europe and America consumed 330,000 tons of coffee, whilst the whole production of the world was 312,000 tons. The present year's estimates are made of consumption 337,000, whilst possible produce is only 274,000 tons. Sugars are well known to be freely mingled with dirt, principally sand, and most of our valuable medicines are so closely imitated and adulterated, as to lose at times their desired efficiency. Quinine and creasote are generally found more or less impure from their adulterations, with a host of others useless to mention. The profession could aid any wholesome reformations from the immense influence they could command by well directed efforts, and this the common enemy of man, should be opposed from every quarter. B.

